# Associations of Dietary Inflammatory Index With Prediabetes and Insulin Resistance

**DOI:** 10.3389/fendo.2022.820932

**Published:** 2022-02-17

**Authors:** Yanling Shu, Xiaocong Wu, Jiating Wang, Xiang Ma, Huawen Li, Yun Xiang

**Affiliations:** ^1^ Department of Laboratory Medicine, Wuhan Children’s Hospital (Wuhan Maternal and Child Healthcare Hospital), Tongji Medical College, Huazhong University of Science and Technology, Wuhan, China; ^2^ Department of Nutrition and Food Hygiene, School of Public Health, Guangdong Medical University, Dongguan, China; ^3^ Guangdong Provincial Key Laboratory of Food, Nutrition and Health, Department of Epidemiology, School of Public Health, Sun Yat-sen University, Guangzhou, China

**Keywords:** dietary inflammatory index, insulin resistance, prediabetes, diabetes, NHANES

## Abstract

**Background and Aims:**

Previous studies suggested that dietary inflammatory index (DII) was associated with a variety of adverse health conditions. However, less is known about the role of DII in prediabetes and insulin resistance (IR). Therefore, this study aimed to investigate whether DII is associated with prediabetes and IR in American adults.

**Method and Results:**

DII scores were calculated using the average of two 24-hour dietary recalls. Linear regression models were performed to evaluate the associations of DII with markers of Type 2 diabetes (T2D) risk, and the associations of DII with prediabetes and IR were estimated using logistic regression model. The diet of the participants showed an anti-inflammatory potential, with a mean DII score of −0.14 (range: −5.83 to +5.32). After controlling for multiple potential confounders, DII scores were positively associated with fasting plasma glucose (FPG) (*β*: 0.009; 95%CI: 0.005 to 0.012), fasting serum insulin (FSI) (*β*: 0.083; 95%CI: 0.067 to 0.099) and homeostatic model assessment of insulin resistance (HOMA-IR) (*β*: 0.092; 95%CI: 0.075 to 0.109). Participants in the highest tertile of DII score have increased odds of prediabetes (OR: 1.40; 95%CI: 1.17 to 1.69; *P* for trend <0.001) and IR (OR: 1.79; 95%CI: 1.49 to 2.14; *P* for trend <0.001) compared with those in the first tertile of DII score.

**Conclusions:**

This study indicates that DII was positively associated with FPG, FSI, and HOMA-IR, and a more pro-inflammatory diet was related to increased odds of insulin resistant and prediabetes.

## Introduction

Currently, there are about 34.2 million people with diabetes in the US, and the prevalence of diabetes is expected to continue to rise in the future (1). Type 2 diabetes (T2D), the most common type of diabetes, is a leading cause of death and poor health and exerts a large and rapidly increasing burden on the US economy (2).

Prediabetes and insulin resistance (IR), major contributing factors in the development of T2D, were characterized by abnormal glucose metabolism (1, 3, 4). To prevent or delay the development of T2D in early stage, a variety of studies were conducted to identify the associations of modifiable factors (e.g., diet, obesity, and smoking) with prediabetes and IR (5–7). As the most concerned and modifiable factor, dietary intake plays a significant role in human health (5, 8, 9). Findings from prior studies have demonstrated that several pro-inflammatory dietary components (e.g., fat and carbohydrate) were associated with increased risk of IR and also other poor health conditions (8–10), while other anti-inflammatory nutrients (e.g., fiber) may have protective effects on health conditions (11). However, it may be difficult to capture the overall effects of diet on health by studying the relationship of single nutrient with diseases. In this context, the dietary inflammatory index (DII) has been proposed to evaluate the inflammatory potential of the overall diet and link diet to inflammation which was a key driver in the progression of prediabetes and IR (12, 13). The DII score is obtained from the diet of an individual based on the pro- and anti-inflammatory properties of the overall dietary compositions, namely, macronutrients, micronutrients, and some other dietary constituents. Previous studies (namely, the studies performed in NHANES) have shown that DII scores were related to a host of health conditions in the general population, namely, hypertension, cancer, and even death (14, 15). However, the associations of DII with prediabetes and IR have received little attention, and several limited studies on the association of DII with markers of Type 2 diabetes (T2D) risk have yielded conflicting results (16–19).

Thus, the main objective of present study was to investigate the associations of the DII with IR and prediabetes in a national representative population in America. The second aim of this study was to investigate the associations between DII and the markers of T2D risk, namely, fasting plasma glucose [FPG], glycohemoglobin [HbA1c], fasting serum insulin [FSI], and homeostatic model assessment of insulin resistance index [HOMA-IR].

## Methods

### Study Participants

The data of current study were extracted from 2007 to 2016 cycles of the National Health and Nutrition Examination Surveys (NHANES), which are ongoing, stratified, multistage probability surveys of the non-institutionalized civilian population residing in the 50 states and districts of Columbia in the United States. The survey consists of questionnaires administered in the home, followed by a standardized health examination in specially equipped mobile examination centers. Beginning in 1999, most data in this nationally representative survey have been released online in 2-year cycles. The NHANES procedures were approved by the National Center for Health Statistics research ethics review board, and the informed consent was obtained from all participants prior to their inclusion in the surveys. The official website provides more detailed information on the methods and protocols of the NHANES (http://www.cdc.gov/nchs/nhanes.htm).

During the 2007–2016 cycles of the NHANES, a total of 30,724 adults constituted the study sample. Due to physiological and pathological factors, namely, pregnancy, diabetes diagnosis, medication use and fasting status, the assessment of the markers of T2D risk or dietary intake might be potentially biased because of the changed dietary habits or other reasons. Therefore, the exclusion criteria of this study were as follows: a) pregnant women (n = 307); b) participants with known diabetes (n = 3,999, self-reported diabetes or use of insulin or oral glucose-lowering medications); c) those with missing data on FPG, HbA1c, and FSI (n = 15,220); d) those with attending the morning examination after fasting <8 h (n = 748); e) those whose dietary recall status is unreliable or does not meet the minimum criteria (n = 1,723); then, 8,727 remained in the study. After further excluding those with missing data on key covariates including sex, educational attainment, race/ethnicity, body mass index (BMI), family poverty income ratio (PIR), hypertension, serum cotinine, and physical activity (n = 801), finally, a total of 7,926 participants (weighted n = 159,386,984) were enrolled in the present study. The results of comparisons among the included participants and those adults without missing data on T2D risk markers and all participants are shown in **Table S1**. Notably, during the examinations of FPG & FSI in the NHANES, there were a series of exclusion criteria (e.g., fasting status, taking insulin or oral medications for diabetes, refusing phlebotomy), resulting in nearly half of the participants missed FPG and FSI.

### Assessment of DII

The development and validation of the DII has previously been reported elsewhere (12, 20). Briefly, dietary intake data for each study participant was first linked to a database that provides a reference global daily mean and standard deviation intake for a total of 45 food parameters from 11 populations around the world. A z-score was derived by subtracting the mean of the database and dividing this value by the standard deviation of the parameter. These z-scores were converted to percentile scores and then centering by doubling and subtracting 1 (from −1 to +1 and centered on 0). Afterwards, each centered proportion was multiplied by the corresponding literature-derived inflammatory effect score for each food parameter. Finally, the overall DII score for each individual is the sum of each food parameter-specific DII score (12).

In this study, dietary data used to calculate the DII score was evaluated by the average of two 24-h dietary recalls. In brief, through face-to-face interview (the first 24-h dietary recall interview) and telephone interview (the second 24-h dietary recall interview), individual foods/beverage consumed during a 24-hour time period before the interview was obtained. The United States Department of Agriculture Food and Nutrient Database for Dietary Studies was used to quantify specific nutrients in the reported dietary components (21). In the NHANES, 31 of the 45 food parameters were available for DII calculation: carbohydrates; protein; fiber; fat; cholesterol; saturated, monounsaturated, and polyunsaturated fatty acids; omega-3 and omega-6 polyunsaturated fatty acids; magnesium; iron; zinc; selenium; niacin; vitamins A, B1, B2, B6, B12, C, D, E; beta carotene; folic acid; alcohol; caffeine; garlic; onion; ginger; and pepper. The unavailable food parameters included eugenol, saffron, trans fat, turmeric, green/black tea, flavan-3-ol, flavones, flavonols, flavonones, anthocyanidins, isoflavones, thyme/oregano and rosemary. To control for the effect of total energy intake, the energy adjusted DII scores were calculated per 1,000 calories of food consumed (using the energy-standardized version of the world database). Given that a significant association between DII and c-reactive protein (CRP) was reported in prior study of the NHANES (22), higher DII (i.e., more positive) scores tend to indicate more pro-inflammatory diets and more negative values tend to indicate more anti-inflammatory (12).

### Measurements of the Markers of T2D Risk

FPG was measured by an enzyme hexokinase method. FSI was measured using an immunoassay method. HbA1c from whole blood was measured using the boronate affinity high performance liquid chromatography system. The concentrations of FPG, FSI, and HbA1c were adjusted for differences in laboratory methodology in the NHANES 2007–2016. The IR status was measured by HOMA-IR, and the 75th percentile value of HOMA-IR was used as a cut-off level to define the IR in present study (23). The corresponding cutoff value was 3.475 for the present study. The HOMA-IR scores were computed as follows: HOMA-IR = [fasting insulin (μU/ml) × fasting glucose (mmol/L)]/22.5 (24). Prediabetes was defined by the HbA1c level of 5.7 to 6.4% and/or impaired fasting glucose level (100–125 mg/dl) and/or impaired glucose tolerance (140–199 mg/dl). In the present study, we also defined undiagnosed diabetes by the HbA1c (≥6.5%) and/or fasting glucose (≥126 mg/dl) and/or impaired glucose tolerance (≥200 mg/dl) (25).

### Study Covariates

Potential confounders, namely, age, sex, ethnicity, education levels, PIR, total energy intake, serum cotinine concentration, BMI, physical activity, hypertension, and diseases history were ascertained using questionnaires or physiological and biochemical tests. The sex was categorized as male and female. The ethnicity was categorized as non-Hispanic white, non-Hispanic black, Mexican American, other Hispanic, and other race. Educational attainment was classified into lower than high school, high school, and higher than high school. BMI was defined as weight divided by height squared (kg/m^2^) (26). PIR was calculated by dividing family (or individual) income by the poverty guidelines specific to the survey year. Physical activity, reported using the Global Physical Activity Questionnaire, was computed as the total metabolic equivalent scores (METs, in minutes/week) summed from the recommended MET scores for each activity. Hypertension was defined as a self-reported history of hypertension, self-reported use of blood pressure medications, systolic blood pressure ≥140 mmHg or diastolic blood pressure ≥90 mmHg. Participants were asked by trained interviewers using the computer-assisted personal interviewing system to obtain health conditions including cardiovascular disease (CVD) diagnosis and cancer diagnosis.

### Statistical Analyses

Categorical variables were expressed as counts (percentages) and were compared using the chi-squared test. Continuous variables were expressed as mean (standard error, SE) if data presented a normal distribution and were compared using analysis of variance (ANOVA), or expressed as medians (25th–75th) if data presented a skewed distribution and were compared by the Wilcoxon rank sum test. FPG, FSI, HbA1c, and HOMA-IR were log-transformed to better approximate a normal distribution. The associations of DII with FPG, FSI, HbA1c, and HOMA-IR were estimated using linear regression model, and the associations of DII with IR and prediabetes were estimated using logistic regression model. Three models were applied in the present study, with adjustment for potential confounders ascertained based on prior publications (27–31) Model 1 was adjusted for energy intake and age. Model 2 was adjusted for energy intake, age, sex, ethnicity, education levels and PIR. Model 3 was further adjusted for serum cotinine concentration, BMI, hypertension, CVD, cancer and physical activity. Notably, participants with undiagnosed diabetes were excluded in models when investigating the relationship between DII and prediabetes. Moreover, to demonstrate the complex interrelationships among the variables of interest, a path analysis with prediabetes status and IR status as the outcomes of interest was carried out and shown in **Figure S1**.

Since previous studies have reported significant associations of obesity with prediabetes and IR (32, 33), a subgroup analysis was performed to assess the potential modification effect by obesity. Obesity was defined as BMI ≥30 kg/m^2^ for the present study. Thus, the present study subjects were classified into two subgroups (obesity: BMI ≥30 kg/m^2^; non-obesity: BMI <30 kg/m^2^). As an interaction term, DII × Obesity, was inserted in the regression models to obtain *P-*value for interaction. In addition, the restricted cubic spline models were applied to evaluate the dose–response relationships of DII with prediabetes and IR in all the participants and subgroups, with adjustment for abovementioned covariates.

To evaluate the robustness of the associations of DII with IR and prediabetes, we further performed a sensitive analysis with adjustment for white blood cell count to control the circulating level of inflammatory marker. Additionally, we performed another sensitive analysis with strict criteria to define prediabetes (HbA1c (6.0–6.5%) and/or FPG (110–125 mg/dl)). Lastly, because there were participants with undiagnosed diabetes in the present study, we also performed an analysis to investigate the association between DII and undiagnosed diabetes.

Survey weighing procedures accounted for the effects of stratification and clustering used in the NHANES study design. We constructed fasting subsample weights for analysis as appropriate to obtain US nationally representative estimates. All data cleaning and statistical analyses were performed using R software (version 3.6.3) (34).

## Results

**Table 1** presents the characteristics of the study participants according to the tertiles of DII. A total of 7,926 adults (weighted n = 159,386,984) were included in the present study. The DII scores of all the participants ranged from −5.83 to 5.32, with a mean of −0.14. Subjects with higher DII scores were younger, males, and more likely to have higher BMI.

**Table 1:** Demographic characteristics of adults aged 18 years and older according to the tertiles of dietary inflammatory index (National Health and Nutrition Examination Survey 2007–2016).

Characteristics	DII			*P*	*P for trend*
	T1 (−5.83, −1.04)	T2 (−1.05, 0.76)	T3 (0.77, 5.32)		
Age, years, mean (SE^a^)	49.8 (0.5)	46.2 (0.4)	40.6 (0.4)	<0.001	<0.001
BMI^b^, kg/m^2^, mean (SE)	27.6 (0.2)	28.8 (0.2)	29.1 (0.2)	<0.001	<0.001
BMI <30	1858 (70.3)	1680 (63.6)	1674 (63.4)		
BMI ≥30	784 (29.7)	962 (36.4)	968 (36.6)		
PIR^c^, mean (SE)	3.4 (0.1)	3 (0.1)	2.6 (0.1)	<0.001	<0.001
Serum cotinine^d^, ng/ml, median (25th–75th)	0 (0–0.1)	0 (0–1.5)	0.2 (0–166)	<0.001	<0.001
Sex, n(%)				<0.001	<0.001
Male	1,043 (39.5)	1,290 (48.8)	1,439 (54.5)		
Female	1,599 (60.5)	1,352 (51.2)	1,203 (45.5)		
Education levels, n(%)				<0.001	<0.001
<high school	440 (16.7)	538 (20.4)	604 (22.9)		
high school	433 (16.4)	648 (24.5)	888 (33.6)		
>high school	1,769 (67)	1,456 (55.1)	1,150 (43.5)		
Ethnicity/Race, n(%)				<0.001	<0.001
Non-Hispanic White	1,253 (47.4)	1,229 (46.5)	1,251 (47.4)		
Non-Hispanic Black	340 (12.9)	419 (15.9)	658 (24.9)		
Hispanic	289 (10.9)	267 (10.1)	226 (8.6)		
Mexican American	364 (13.8)	460 (17.4)	357 (13.5)		
Other Race	396 (15)	267 (10.1)	150 (5.7)		
Physical activity (MET)^e^, median (25th–75th)	1,440 (360–3600)	1,440 (240–4091)	1,560 (240–5760)	0.02	0.80
Hypertension^f^, yes, n (%)	1,020 (38.6)	971 (36.8)	877 (33.2)	<0.001	<0.001
CVD^g^, ever, n (%)	220 (8.3)	231 (8.7)	194 (7.3)	0.16	0.191
Cancer, ever, n (%)	290 (11)	213 (8.1)	162 (6.1)	<0.001	<0.001

a SE, standard error.

b BMI, body mass index.

c PIR, family poverty income ratio.

d Serum cotinine, a metabolite of nicotine, is a marker of exposure to tobacco smoke.

e MET, metabolic equivalents.

f Hypertension was defined as a self-reported history of hypertension, self-reported use of blood pressure medications, systolic blood pressure ≥140 mmHg or diastolic blood pressure ≥90 mmHg.

g CVD, cardiovascular diseases including heart failure, coronary heart disease, angina, heart attack, stroke.

**Table 2** shows the associations between DII and the markers of T2D risk. The associations of continuous DII scores with FPG, FSI, and HOMA-IR were significant after adjustment for potential confounders, but the relationship between DII and HbA1c was significant only after adjustment for covariates in model 2. After fully adjustment for covariates in model 3, participants in the second or the third tertile of DII score had higher levels of FPG, FSI, and HOMA-IR than those in the first tertile of DII score, and the corresponding *β* coefficients (95% CI) for the highest tertile of DII score were 0.021 (95%CI: 0.013 to 0.028), 0.191 (95%CI: 0.153 to 0.229), and 0.212 (95%CI: 0.172 to 0.252) for FPG, FSI, and HOMA-IR, respectively.

**Table 2:** *β*-Coefficients (95%CIs)^a^ for the relationship between the dietary inflammatory index and the markers of T2D risk (National Health and Nutrition Examination Survey 2007–2016).

Outcomes	*β* (95% *CI*)				*P* for trend
	Per SD^b^ increase	T1 (−5.83, −1.04)	T2 (−1.05, 0.76)	T3 (0.77, 5.32)	
FPG^c^					
Model 1^g^	0.004 (0.001, 0.007)	ref	0.019 (0.010, 0.027)	0.012 (0.004, 0.019)	0.003
Model 2^h^	0.010 (0.007, 0.013)	ref	0.021 (0.014, 0.029)	0.025 (0.017, 0.033)	<0.001
Model 3^i^	0.009 (0.005, 0.012)	ref	0.017 (0.010, 0.025)	0.021 (0.013, 0.028)	<0.001
FSI^d^					
Model 1^g^	0.099 (0.081, 0.117)	ref	0.178 (0.135, 0.221)	0.240 (0.198, 0.282)	<0.001
Model 2^h^	0.098 (0.079, 0.116)	ref	0.167 (0.125, 0.209)	0.230 (0.184, 0.276)	<0.001
Model 3^i^	0.083 (0.067, 0.099)	ref	0.122 (0.089, 0.155)	0.191 (0.153, 0.229)	<0.001
HbA1C^e^					
Model 1^g^	−0.000 (−0.003, 0.002)	ref	0.006 (0.000, 0.012)	0.000 (-0.005, 0.006)	0.941
Model 2^h^	0.004 (0.002, 0.006)	ref	0.009 (0.004, 0.014)	0.010 (0.005, 0.015)	<0.001
Model 3^i^	0.002 (−0.000, 0.004)	ref	0.006 (0.001, 0.011)	0.005 (0.000, 0.010)	0.033
HOMA-IR^f^					
Model 1^g^	0.104 (0.085, 0.122)	ref	0.197 (0.150, 0.243)	0.251 (0.209, 0.294)	<0.001
Model 2^h^	0.108 (0.089, 0.126)	ref	0.188 (0.144, 0.232)	0.255 (0.207, 0.302)	<0.001
Model 3^i^	0.092 (0.075, 0.109)	ref	0.139 (0.105, 0.174)	0.212 (0.172, 0.252)	<0.001

a CI, confidence interval.

b SD, standard deviation.

c FPG, fasting plasma glucose.

d FSI, fasting serum insulin.

e HbA1c, glycohemoglobin.

f HOMA-IR, homeostatic model assessment of insulin resistance.

g Model 1 adjusted for energy (continuous) and age (continuous).

h Model 2 adjusted for energy (continuous), age (continuous), sex (categorical), education levels (categorical), ethnicity/race (categorical), PIR (continuous).

i Model 3 adjusted for energy (continuous), age (continuous), sex (categorical), education levels (categorical), ethnicity/race (categorical), PIR (continuous), BMI (categorical), hypertension (categorical), CVD (categorical), cancer (categorical), serum cotinine (continuous), and physical activity (continuous).

The relationship between DII and prediabetes according to the tertiles of DII score is listed in **Table 3**. Compared with the individuals in the lowest tertile of DII, the multivariable-adjusted odds ratios (95%CIs) of prediabetes for the second and the third tertiles of DII was 1.26 (95%CI: 1.08 to 1.47) and 1.40 (95%CI: 1.17 to 1.69), respectively (*P* for trend <0.001). In the results of subgroup analyses, the association between DII and prediabetes was more evident among participants with obesity (OR:1.50; 95%CI:1.04 to 2.15), but there was no significant modification effect of BMI on the DII-prediabetes association (*P* value for interaction = 0.904).

**Table 3:** Multivariable-adjusted odds ratios (95%CIs)^a^ of prediabetes according to the tertiles of dietary inflammatory index (National Health and Nutrition Examination Survey 2007–2016).

Models	OR^b^ (95%CI)				*P* for trend	*P* for interaction^h^
	Per SD^c^ increase	T1 (−5.83, −1.04)	T2 (−1.05, 0.76)	T3 (0.77, 5.32)		
Overall						
Model 1^e^	1.25 (1.17, 1.34)	ref	1.44 (1.25, 1.66)	1.68 (1.44, 1.97)	<0.001	
Model 2^f^	1.20 (1.12, 1.29)	ref	1.32 (1.14, 1.53)	1.49 (1.25, 1.78)	<0.001	**-**
Model 3^g^	1.17 (1.09, 1.26)	ref	1.26 (1.08, 1.47)	1.40 (1.17, 1.69)	<0.001	
BMI^d^ ≥30 kg/m^2^						
Model 1^e^	1.19 (1.04, 1.35)	ref	1.51 (1.15, 1.97)	1.44 (1.05, 1.97)	0.036	
Model 2^f^	1.20 (1.04, 1.39)	ref	1.48 (1.13, 1.95)	1.46 (1.03, 2.06)	0.053	
Model 3^g^	1.21 (1.05, 1.41)	ref	1.52 (1.14, 2.02)	1.50 (1.04, 2.15)	0.044	
BMI^d^ <30 kg/m^2^						0.904
Model 1^e^	1.24 (1.15, 1.34)	ref	1.33 (1.09, 1.61)	1.67 (1.38, 2.00)	<0.001	
Model 2^f^	1.16 (1.07, 1.26)	ref	1.19 (0.98, 1.45)	1.41 (1.17, 1.70)	<0.001	
Model 3^g^	1.16 (1.07, 1.26)	ref	1.18 (0.97, 1.43)	1.39 (1.15, 1.67)	0.001	

a CI, confidence interval.

b OR, odds ratio.

c SD, standard deviation.

d BMI, body mass index.

e Model 1 adjusted for energy intake (continuous) and age (continuous).

f Model 2 adjusted for energy intake (continuous), age (continuous), sex (categorical), education levels (categorical), ethnicity/race (categorical), PIR (continuous).

g Model 3 adjusted for energy intake (continuous), age (continuous), sex (categorical), education levels (categorical), ethnicity/race (categorical), PIR (continuous), BMI (categorical, only for overall population), hypertension (categorical), CVD (categorical), cancer (categorical), serum cotinine (continuous), and physical activity (continuous).

h P-value for interaction of the Model 3.

The association between DII and IR according to the tertiles of DII score is listed in **Table 4**. An increment of 1 SD in the DII score (i.e., 1.91 units) was associated with a 26% higher odds of IR (OR: 1.26; 95%CI: 1.18 to 1.36) after adjustment for potential covariates. Additionally, we also assessed the relationship between tertiles of DII score and IR. Compared with the individuals in the lowest tertile of DII, the odds of IR increased by 79% among the individuals in the highest tertile of DII score (OR: 1.79; 95%CI: 1.49 to 2.14). In analysis stratified by BMI, participants with obesity in the highest tertile of DII had an 79% higher odds of IR than those with DII score in the lowest tertile (OR:1.79; 95%CI:1.38 to 2.34). Similarly, participants without obesity in the highest DII tertile had a 78% higher odds of IR than those with DII score in the lowest tertile (OR: 1.78; 95%CI: 1.34 to 2.37). However, the modification effect of BMI on the DII-IR association was insignificant (*P-value for interaction* = 0.653).

**Table 4:** Multivariable-adjusted odds ratios (95%CIs)^a^ of IR according to the tertiles of dietary inflammatory index (National Health and Nutrition Examination Survey 2007–2016).

Models	OR^b^ (95%CI)				*P* for trend	*P* for interaction^h^
	Per SD^c^ increase	T1 (−5.83, −1.04)	T2 (−1.05, 0.76)	T3 (0.77, 5.32)		
Overall						
Model 1^e^	1.25 (1.19, 1.32)	ref	1.59 (1.37, 1.86)	1.80 (1.56, 2.08)	<0.001	
Model 2^f^	1.27 (1.20, 1.35)	ref	1.58 (1.36, 1.85)	1.86 (1.56, 2.20)	<0.001	**-**
Model 3^g^	1.26 (1.18, 1.36)	ref	1.46 (1.23, 1.75)	1.79 (1.49, 2.14)	<0.001	
BMI^d^ ≥30 kg/m^2^						
Model 1^e^	1.17 (1.06, 1.29)	ref	1.33 (1.04, 1.70)	1.60 (1.25, 2.07)	<0.001	
Model 2^f^	1.19 (1.08, 1.32)	ref	1.34 (1.05, 1.71)	1.69 (1.31, 2.18)	<0.001	
Model 3^g^	1.22 (1.10, 1.36)	ref	1.36 (1.06, 1.74)	1.79 (1.38, 2.34)	<0.001	
BMI^d^ <30 kg/m^2^						0.653
Model 1^e^	1.37 (1.25, 1.50)	ref	1.77 (1.31, 2.38)	2.05 (1.56, 2.68)	<0.001	
Model 2^f^	1.33 (1.21, 1.46)	ref	1.64 (1.22, 2.22)	1.81 (1.37, 2.40)	<0.001	
Model 3^g^	1.32 (1.19, 1.46)	ref	1.62 (1.19, 2.20)	1.78 (1.34, 2.37)	<0.001	

a CI, confidence interval.

b OR, odds ratio.

c SD, standard deviation.

d BMI, body mass index.

e Model 1 adjusted for energy intake (continuous) and age (continuous).

f Model 2 adjusted for energy intake (continuous), age (continuous), sex (categorical), education levels (categorical), ethnicity/race (categorical), PIR (continuous).

g Model 3 adjusted for energy intake (continuous), age (continuous), sex (categorical), education levels (categorical), ethnicity/race (categorical), PIR (continuous), BMI (categorical, only for overall population), hypertension (categorical), CVD (categorical), cancer (categorical), serum cotinine (continuous), and physical activity (continuous).

h P-value for interaction of the Model 3.

**Figure 1:**
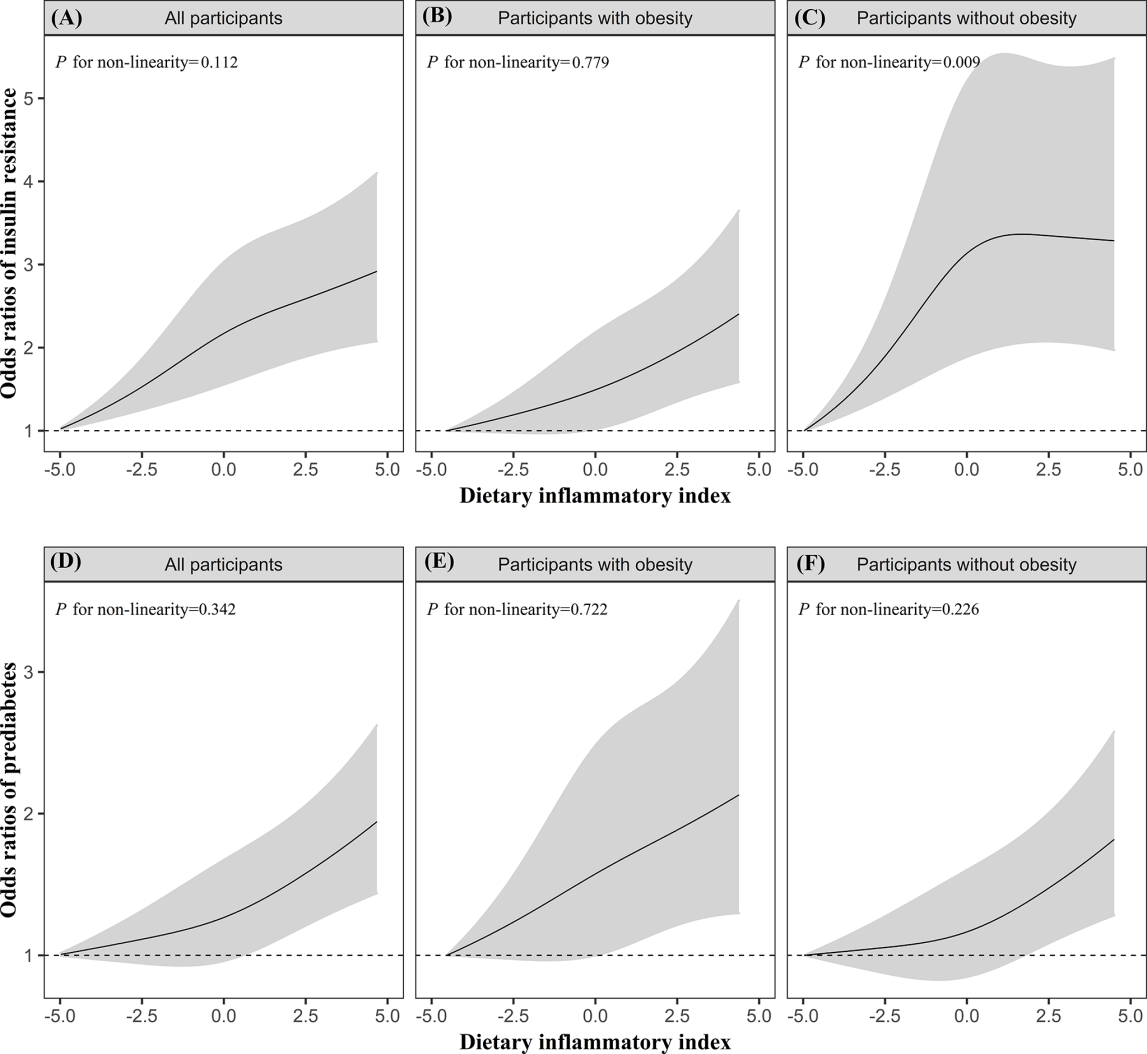
The dose–response relationships of DII with IR **(A–C)** and prediabetes **(D–F)** in all participants, participants with obesity, participants without obesity. Results were from restricted cubic spline models. Models were adjusted for energy intake (continuous), age (continuous), sex (categorical), education levels (categorical), ethnicity/race (categorical), PIR (continuous), BMI (categorical, only for overall population), hypertension (categorical), CVD (categorical), cancer (categorical), serum cotinine (continuous), and physical activity (continuous).

Additionally, the dose–response relationships of DII with prediabetes and IR were further assessed by using the restricted cubic splines. Overall, there was a linear dose–response relationship between DII score and IR (*P* for non-linearity = 0.112) and prediabetes (*P* for non-linearity = 0.342), but a significant non-linear association of DII with IR was observed in the participants without obesity (*P* for non-linearity = 0.009; **Figure 1**).

The results of sensitive analyses are shown in **Tables S2–S4**. Specifically, the significant associations of DII with IR and prediabetes were persisted after adjusting for white blood cell count to control the circulating level of inflammatory marker. The relationship between DII and prediabetes was substantially unchanged in models with strict criteria to define prediabetes. In addition, a total of 690 participants with undiagnosed diabetes were identified, and we observed a significant relationship between DII and increased odds of undiagnosed diabetes (OR:1.31; 95%CI: 1.16 to 1.50).

## Discussion

In this cross-sectional study of American adults, DII was significantly associated with increased levels of FPG, FSI, and HOMA-IR, and was associated with the increased odds of IR and prediabetes after adjustment for potential confounders. Similar findings yielded in analysis stratified by BMI. Besides, restricted cubic spline models showed linear dose–response relationships between DII and the odds of insulin resistant and prediabetes.

The DII score computed from 31 food parameters, ranged from −5.83 to +5.32 with a mean of −0.14, which indicates an anti-inflammatory diet. These results were consistent with prior studies conducted in Mexico, Spain, Italy, and France (35–39) Notably, for this study, subjects who were older, female, and those with hypertension were more likely to have anti-inflammatory diet, which was similar to a case-control study from Bahrain (40) The exact reason for these observed findings is unclear, we speculated that those adults might be more likely to pay more attention to what they are eating and stay healthy.

In the present study, we found significant associations of DII with the markers of T2D risk (i.e., FPG, FSI, and HOMA-IR), IR, and prediabetes, suggesting an important role of diet on the homeostasis of glucose metabolism. Compared with previous studies, findings from the present study differed from several earlier studies (17, 19, 41), which reported non-significant associations of DII with the markers of T2D risk and IR in more homogeneous study populations. These conflicting results may be partly due to the differences in sample size, study population, and region representation. For instance, there were only 1,352 participants in the Luxembourg study (19), while the present study included 7,926 participants for primary analysis. In the Iranian study, the participants that were residents of district 13 of Tehran, were not a representative sample of Iranian populations (17). The Brazil study included only young adults aged 23–25 years (41), while the present study included both men and women aged 18 years or older. However, the findings of the present study were in line with the results of a Dutch cohort study, which showed that the DII was positively associated with IR and fasting glucose concentration (16). Additionally, in a more recent cross-sectional study conducted in South Africa, DII was positively associated with fasting glucose, insulin and HOMA-IR (18). Only one study reported the correlation between DII and prediabetes, suggesting that subjects who consumed a pro-inflammatory diet were at increased risk of prediabetes compared with those who consumed a more anti-inflammatory diet (42), whereas, the abovementioned three studies were equally small in sample size. The present research seems to overcome the above mentioned shortcomings and may add new evidence to the limited literature to elucidate the role of DII on the markers of T2D risk, prediabetes, and IR.

Biologic mechanisms underlying the associations of DII with prediabetes and IR remain unclear. Nonetheless, previous studies suggested that pro-inflammatory diet could promote low-grade inflammation (43) that is characterized by elevated pro-inflammatory markers, namely, CRP, TNF-α, and interleukin-6 which may lead to prediabetes and IR (44–46). Furthermore, high intakes of saturated fat and animal protein (47), which are the dietary constituents for DII calculation, may have adverse effects on prediabetes and IR (48). Lastly, studies in the past found that higher DII scores could promote the development of obesity that further contributed to the development of prediabetes and IR (49, 50). However, the present results showed that the associations of DII with IR and prediabetes were significant, with or without adjustment for BMI, indicating the associations of DII with IR and prediabetes might be independent from BMI. In addition, in the stratified models, the present study demonstrated significant associations of DII with IR and prediabetes in participants with or without obesity, and the interaction of DII and BMI was not found to reach statistical significance, implying that the effect of DII on IR and prediabetes might be homogenous in participants with or without obesity. In view of above plausible reasons, future longitudinal cohort and experimental studies should be carried out to reveal the specific causal mechanisms. Furtherly, participants with prediabetes or IR were more likely to develop diabetes and CVD (51, 52), and previous studies have shown that pro-inflammatory diet is associated with increased risk of diabetes and CVD (14, 53). The findings of the present study may further add new evidence to explain their interconnectedness and add new insights into the mechanisms of the occurrence and development of diabetes and CVD.

The present study was based on a large nationally representative survey, which allowed to adjust for multiple covariates and increased the statistical power of the findings. Despite its strengths, we also acknowledge that this study was subject to some limitations. First, the temporal or causal relationships cannot be inferred because of the cross-sectional design for NHANES. However, the study participants were free from known diabetes, so they would not change their diet due to the diagnosis, and thus the dietary information collected at the same time when blood samples were collected could reflect participants’ usual dietary habits. Second, although multicovariates were included in models, we could not exclude the residual confounding effect from unmeasured or unavailable covariates (e.g., genetic factors and use of anti-inflammatory medications). Third, only 31 out of 45 parameters were extracted from 24-hour recalls due to the questionnaire setting. However, previous studies have shown that there was no degradation in predictive ability of the DII in calculation using only 27 or 28 parameters (54, 55). Fourth, although two days of dietary recall were used to reduce the measurement bias, dietary intake of individuals estimated by the average of two 24-hour dietary recalls instead of the NCI method (a useful method to reduce within-person variation and produce a more precise estimate when using NHANES dietary data) does not account for day-to-day variations in diet or seasonal variability in diet patterns. Fifth, subjects included in the present study are more likely to be those apparently healthy adults, which may limit the generalizability of these findings. Lastly, participants with missing data on covariates were excluded, which may limit the generalizability of these findings.

## Conclusions

In conclusion, the present study suggests that DII was positively associated with FPG, FSI, and HOMA-IR, and a more pro-inflammatory diet (expressed as a higher DII score) may increase the odds of insulin resistant and prediabetes. Therefore, the promotion of anti-inflammatory diet might maintain glucose–insulin homeostasis, and subsequently lower the odds of insulin resistant and prediabetes.

## Data Availability Statement

The raw data supporting the conclusions of this article will be made available by the authors, without undue reservation.

## Ethics Statement

The studies involving human participants were reviewed and approved by the National Center for Health Statistics research ethics review board. The patients/participants provided their written informed consent to participate in this study.

## Author Contributions

YS, XW, JW, XM, HL, and YX conceived and designed the research. YS wrote the manuscript draft. YS performed the statistical analysis. YS, XW, JW, and XM participated in revising the manuscript. HL and YX provided content and feedback to the manuscript, reviewed and edited. All authors contributed to the article and approved the submitted version.

## Funding

This research was funded by the Guangdong Science & Technology Development Project [2013B031800012], and the National Natural Science Foundation of China [81874260].

## Conflict of Interest

The authors declare that the research was conducted in the absence of any commercial or financial relationships that could be construed as a potential conflict of interest.

The reviewer YBZ declared a shared affiliation with several of the authors, YS, XM, YX, to the handling editor at time of review.

## Publisher’s Note

All claims expressed in this article are solely those of the authors and do not necessarily represent those of their affiliated organizations, or those of the publisher, the editors and the reviewers. Any product that may be evaluated in this article, or claim that may be made by its manufacturer, is not guaranteed or endorsed by the publisher.

## References

[1] Prevention CfDCa National Diabetes Statistics Report (2020). Available at: https://www.cdc.gov/diabetes/pdfs/data/statistics/national-diabetes-statistics-report.pdf (Accessed 03/05).

[2] American Diabetes Association. Economic Costs of Diabetes in the U.S. @ in 2012. Diabetes Care (2013) 36(4):1033–46. doi: 10.2337/dc12-2625 PMC3609540 23468086

[3] National Institute of Diabetes and Digestive and Kidney Diseases Diseases NIoDaDaK Insulin Resistance & Prediabetes. Available at: https://www.niddk.nih.gov/health-information/diabetes/overview/what-is-diabetes/prediabetes-insulin-resistance (Accessed 2021/03/08).

[4] Antuna-Puente B Disse E Rabasa-Lhoret R Laville M Capeau J Bastard JP. How Can We Measure Insulin Sensitivity/Resistance? Diabetes Metab (2011) 37(3):179–88. doi: 10.1016/j.diabet.2011.01.002 21435930

[5] Mirabelli M Chiefari E Arcidiacono B Corigliano DM Brunetti FS Maggisano V. Mediterranean Diet Nutrients to Turn the Tide Against Insulin Resistance and Related Diseases. Nutrients (2020) 12(4):1066. doi: 10.3390/nu12041066 PMC7230471 32290535

[6] Miao Z Alvarez M Ko A Bhagat Y Rahmani E Jew B. The Causal Effect of Obesity on Prediabetes and Insulin Resistance Reveals the Important Role of Adipose Tissue in Insulin Resistance. PloS Genet (2020) 16(9):e1009018. doi: 10.1371/journal.pgen.1009018 32925908 PMC7515203

[7] Reaven G Tsao PS. Insulin Resistance and Compensatory Hyperinsulinemia: The Key Player Between Cigarette Smoking and Cardiovascular Disease? J Am Coll Cardiol (2003) 41(6):1044–7. doi: 10.1016/S0735-1097(02)02982-0 12651055

[8] Tricò D Trifirò S Mengozzi A Morgantini C Baldi S Mari A. Reducing Cholesterol and Fat Intake Improves Glucose Tolerance by Enhancing β Cell Function in Nondiabetic Subjects. J Clin Endocrinol Metab (2018) 103(2):622–31. doi: 10.1210/jc.2017-02089 29095990

[9] Riccardi G Giacco R Rivellese AA. Dietary Fat, Insulin Sensitivity and the Metabolic Syndrome. Clin Nutr (2004) 23(4):447–56. doi: 10.1016/j.clnu.2004.02.006 15297079

[10] Lee CL Liu WJ Wang JS. Associations of Low-Carbohydrate and Low-Fat Intakes With All-Cause Mortality in Subjects With Prediabetes With and Without Insulin Resistance. Clin Nutr (2020) 40(5):3601–7. doi: 10.1016/j.clnu.2020.12.019 33390277

[11] Tucker LA. Fiber Intake and Insulin Resistance in 6374 Adults: The Role of Abdominal Obesity. Nutrients (2018) 10(2):237. doi: 10.3390/nu10020237 PMC5852813 29461482

[12] Shivappa N Steck SE Hurley TG Hussey JR Hebert JR. Designing and Developing a Literature-Derived, Population-Based Dietary Inflammatory Index. Public Health Nutr (2014) 17(8):1689–96. doi: 10.1017/S1368980013002115 PMC3925198 23941862

[13] Weaver JR Odanga JJ Breathwaite EK Treadwell ML Murchinson AC Walters G. An Increase in Inflammation and Islet Dysfunction Is a Feature of Prediabetes. Diabetes Metab Res Rev (2020) 37(6):e3405. doi: 10.1002/dmrr.3405 33463010

[14] Vissers LET Waller M van der Schouw YT Hebert JR Shivappa N Schoenaker D. A Pro-Inflammatory Diet Is Associated With Increased Risk of Developing Hypertension Among Middle-Aged Women. Nutr Metab Cardiovasc Dis (2017) 27(6):564–70. doi: 10.1016/j.numecd.2017.03.005 28446366

[15] Deng FE Shivappa N Tang Y Mann JR Hebert JR. Association Between Diet-Related Inflammation, All-Cause, All-Cancer, and Cardiovascular Disease Mortality, With Special Focus on Prediabetics: Findings From NHANES III. Eur J Nutr (2017) 56(3):1085–93. doi: 10.1007/s00394-016-1158-4 26825592

[16] van Woudenbergh GJ Theofylaktopoulou D Kuijsten A Ferreira I van Greevenbroek MM van der Kallen CJ. Adapted Dietary Inflammatory Index and Its Association With a Summary Score for Low-Grade Inflammation and Markers of Glucose Metabolism: The Cohort Study on Diabetes and Atherosclerosis Maastricht (CODAM) and the Hoorn Study. Am J Clin Nutr (2013) 98(6):1533–42. doi: 10.3945/ajcn.112.056333 24153342

[17] Moslehi N Ehsani B Mirmiran P Shivappa N Tohidi M Hebert JR. Inflammatory Properties of Diet and Glucose-Insulin Homeostasis in a Cohort of Iranian Adults. Nutrients (2016) 8(11):735. doi: 10.3390/nu8110735 PMC5133119 27869717

[18] Mtintsilana A Micklesfield LK Chorell E Olsson T Shivappa N Hebert JR. Adiposity Mediates the Association Between the Dietary Inflammatory Index and Markers of Type 2 Diabetes Risk in Middle-Aged Black South African Women. Nutrients (2019) 11(6):1246. doi: 10.3390/nu11061246 PMC6628082 31159253

[19] Alkerwi A Shivappa N Crichton G Hebert JR. No Significant Independent Relationships With Cardiometabolic Biomarkers Were Detected in the Observation of Cardiovascular Risk Factors in Luxembourg Study Population. Nutr Res (2014) 34(12):1058–65. doi: 10.1016/j.nutres.2014.07.017 PMC4329249 25190219

[20] Wang T Jiang H Wu Y Wang W Zhang D. The Association Between Dietary Inflammatory Index and Disability in Older Adults. Clin Nutr (2020) 40(4):2285–92. doi: 10.1016/j.clnu.2020.10.017 33121836

[21] Food Surveys Research Group. Food-Surveys-Research-Group Food and Nutrient Database for Dietary Studies. Available at: http://www.ars.usda.gov/nea/bhnrc/fsrg.

[22] Shivappa N Wirth MD Murphy EA Hurley TG Hébert JR. Association Between the Dietary Inflammatory Index (DII) and Urinary Enterolignans and C-Reactive Protein From the National Health and Nutrition Examination Survey-2003-2008. Eur J Nutr (2019) 58(2):797–805. doi: 10.1007/s00394-018-1690-5 29675557

[23] Meigs JB Larson MG Fox CS Keaney JF Jr Vasan RS Benjamin EJ. Association of Oxidative Stress, Insulin Resistance, and Diabetes Risk Phenotypes: The Framingham Offspring Study. Diabetes Care (2007) 30(10):2529–35. doi: 10.2337/dc07-0817 17586736

[24] Wallace TM Levy JC Matthews DR. Use and Abuse of HOMA Modeling. Diabetes Care (2004) 27(6):1487–95. doi: 10.2337/diacare.27.6.1487 15161807

[25] American Diabetes Association. Classification and Diagnosis of Diabetes: Standards of Medical Care in Diabetes-2021. Diabetes Care (2021) 44(Suppl 1):S15–s33. doi: 10.2337/dc21-S002 33298413

[26] National Center for Health Statistics. Plan and Operation of the Third National Health and Nutrition Examination Survey, 1988-94. Series 1: Programs and Collection Procedures. Vital Health Stat (1994) 32):1–407. 7975354

[27] Choi CHJ Cohen P. How Does Obesity Lead to Insulin Resistance? Elife (2017) 6:e33298. doi: 10.7554/eLife.33298 29239299 PMC5730368

[28] Thiering E Bruske I Kratzsch J Thiery J Sausenthaler S Meisinger C. Prenatal and Postnatal Tobacco Smoke Exposure and Development of Insulin Resistance in 10 Year Old Children. Int J Hyg Environ Health (2011) 214(5):361–8. doi: 10.1016/j.ijheh.2011.04.004 21570350

[29] Soleimani M. Insulin Resistance and Hypertension: New Insights. Kidney Int (2015) 87(3):497–9. doi: 10.1038/ki.2014.392 25723632

[30] Fujita H Hosono A Shibata K Tsujimura S Oka K Okamoto N. Physical Activity Earlier in Life Is Inversely Associated With Insulin Resistance Among Adults in Japan. J Epidemiol (2019) 29(2):57–60. doi: 10.2188/jea.JE20170180 30249943 PMC6336722

[31] Hellgren MI Daka B Jansson PA Lindblad U Larsson CA. Insulin Resistance Predicts Early Cardiovascular Morbidity in Men Without Diabetes Mellitus, With Effect Modification by Physical Activity. Eur J Prev Cardiol (2015) 22(7):940–9. doi: 10.1177/2047487314537917 24879358

[32] Schwartz MW Kahn SE. Insulin Resistance and Obesity. Nature (1999) 402(6764):860–1. doi: 10.1038/47209 10622250

[33] Brož J Malinovská J Nunes MA Kučera K Rožeková K Žejglicová K. Prevalence of Diabetes and Prediabetes and Its Risk Factors in Adults Aged 25-64 in the Czech Republic: A Cross-Sectional Study. Diabetes Res Clin Pract (2020) 170:108470. doi: 10.1016/j.diabres.2020.108470 32998019

[34] The R Core Team. Team RC R Version 3.6.3. Available at: https://www.r-project.org/.

[35] Shivappa N Zucchetto A Montella M Serraino D Steck SE La Vecchia C. Inflammatory Potential of Diet and Risk of Colorectal Cancer: A Case-Control Study From Italy. Br J Nutr (2015) 114(1):152–8. doi: 10.1017/S0007114515001828 26050563

[36] Neufcourt L Assmann KE Fezeu LK Touvier M Graffouillère L Shivappa N. Prospective Association Between the Dietary Inflammatory Index and Cardiovascular Diseases in the SUpplémentation En VItamines Et Minéraux AntioXydants (SU.VI.MAX) Cohort. J Am Heart Assoc (2016) 5(3):e002735. doi: 10.1161/JAHA.115.002735 27068628 PMC4943250

[37] Ramallal R Toledo E Martínez-González MA Hernández-Hernández A García-Arellano A Shivappa N. Dietary Inflammatory Index and Incidence of Cardiovascular Disease in the SUN Cohort. PloS One (2015) 10(9):e0135221. doi: 10.1371/journal.pone.0135221 26340022 PMC4560420

[38] Medina-Remon A Casas R Tressserra-Rimbau A Ros E Martinez-Gonzalez MA Fito M. Polyphenol Intake From a Mediterranean Diet Decreases Inflammatory Biomarkers Related to Atherosclerosis: A Substudy of the PREDIMED Trial. Br J Clin Pharmacol (2017) 83(1):114–28. doi: 10.1111/bcp.12986 PMC5338147 27100393

[39] Denova-Gutiérrez E Muñoz-Aguirre P Shivappa N Hébert JR Tolentino-Mayo L Batis C. Dietary Inflammatory Index and Type 2 Diabetes Mellitus in Adults: The Diabetes Mellitus Survey of Mexico City. Nutrients (2018) 10(4):385. doi: 10.3390/nu10040385 PMC5946170 29561774

[40] Jahrami H Faris MA Ghazzawi HA Saif Z Habib L Shivappa N. Increased Dietary Inflammatory Index Is Associated With Schizophrenia: Results of a Case-Control Study From Bahrain. Nutrients (2019) 11(8):1867. doi: 10.3390/nu11081867 PMC6722742 31405205

[41] Carvalho CA Silva AAM Assuncao MCF Fonseca PCA Barbieri MA Bettiol H. The Dietary Inflammatory Index and Insulin Resistance or Metabolic Syndrome in Young Adults. Nutrition (2019) 58:187–93. doi: 10.1016/j.nut.2018.07.014 30504010

[42] Vahid F Shivappa N Karamati M Naeini AJ Hebert JR Davoodi SH. Association Between Dietary Inflammatory Index (DII) and Risk of Prediabetes: A Case-Control Study. Appl Physiol Nutr Metab (2017) 42(4):399–404. doi: 10.1139/apnm-2016-0395 28177734

[43] Shivappa N Bonaccio M Hebert JR Di Castelnuovo A Costanzo S Ruggiero E. Association of Proinflammatory Diet With Low-Grade Inflammation: Results From the Moli-Sani Study. Nutrition (2018) 54:182–8. doi: 10.1016/j.nut.2018.04.004 PMC6138548 29982145

[44] Weisberg SP McCann D Desai M Rosenbaum M Leibel RL Ferrante AW Jr. Obesity Is Associated With Macrophage Accumulation in Adipose Tissue. J Clin Invest (2003) 112(12):1796–808. doi: 10.1172/JCI200319246 PMC296995 14679176

[45] Kim CS Park HS Kawada T Kim JH Lim D Hubbard NE. Circulating Levels of MCP-1 and IL-8 Are Elevated in Human Obese Subjects and Associated With Obesity-Related Parameters. Int J Obes (Lond) (2006) 30(9):1347–55. doi: 10.1038/sj.ijo.0803259 16534530

[46] Kato K Otsuka T Saiki Y Kobayashi N Nakamura T Kon Y. Association Between Elevated C-Reactive Protein Levels and Prediabetes in Adults, Particularly Impaired Glucose Tolerance. Can J Diabetes (2019) 43(1):40–5.e2. doi: 10.1016/j.jcjd.2018.03.007 30026044

[47] Chen Z Franco OH Lamballais S Schoufour JD Muka T. Associations of Specific Dietary Protein With Longitudinal Insulin Resistance, Prediabetes and Type 2 Diabetes: The Rotterdam Study. Clin Nutr (2019) 39(1):242–9. doi: 10.1016/j.clnu.2019.01.021 30739809

[48] Hernandez EA Kahl S Seelig A Begovatz P Irmler M Kupriyanova Y. Acute Dietary Fat Intake Initiates Alterations in Energy Metabolism and Insulin Resistance. J Clin Invest (2017) 127(2):695–708. doi: 10.1172/JCI89444 28112681 PMC5272194

[49] Ruiz-Canela M Zazpe I Shivappa N Hebert JR Sanchez-Tainta A Corella D. Dietary Inflammatory Index and Anthropometric Measures of Obesity in a Population Sample at High Cardiovascular Risk From the PREDIMED (PREvencion Con DIeta MEDiterranea) Trial. Br J Nutr (2015) 113(6):984–95. doi: 10.1017/S0007114514004401 PMC4870040 25720588

[50] Kahn SE Hull RL Utzschneider KM. Mechanisms Linking Obesity to Insulin Resistance and Type 2 Diabetes. Nature (2006) 444(7121):840–6. doi: 10.1038/nature05482 17167471

[51] Cai X Zhang Y Li M Wu JH Mai L Li J. Association Between Prediabetes and Risk of All Cause Mortality and Cardiovascular Disease: Updated Meta-Analysis. BMJ (2020) 370:m2297. doi: 10.1136/bmj.m2297 32669282 PMC7362233

[52] Cai X Liu X Sun L He Y Zheng S Zhang Y. Prediabetes and the Risk of Heart Failure: A Meta-Analysis. Diabetes Obes Metab (2021) 23(8):1746–53. doi: 10.1111/dom.14388 33769672

[53] Tan QQ Du XY Gao CL Xu Y. Higher Dietary Inflammatory Index Scores Increase the Risk of Diabetes Mellitus: A Meta-Analysis and Systematic Review. Front Endocrinol (Lausanne) (2021) 12:693144. doi: 10.3389/fendo.2021.693144 34456864 PMC8385131

[54] Shivappa N Steck SE Hurley TG Hussey JR Ma Y Ockene IS. A Population-Based Dietary Inflammatory Index Predicts Levels of C-Reactive Protein in the Seasonal Variation of Blood Cholesterol Study (SEASONS). Public Health Nutr (2014) 17(8):1825–33. doi: 10.1017/S1368980013002565 PMC3983179 24107546

[55] Park YM Choi MK Lee SS Shivappa N Han K Steck SE. Dietary Inflammatory Potential and Risk of Mortality in Metabolically Healthy and Unhealthy Phenotypes Among Overweight and Obese Adults. Clin Nutr (2019) 38(2):682–8. doi: 10.1016/j.clnu.2018.04.002 PMC6389430 29705061

