# Supplementary material for: Associations of Dietary Inflammatory Index With Prediabetes and Insulin Resistance
Source: Front Endocrinol (Lausanne). 2022 Feb 17;13:820932. doi: 10.3389/fendo.2022.820932 (PMC8892213; doi:10.3389/fendo.2022.820932)
Supplement: Supplementary file 1 [file DataSheet_1.docx]

Supplementary Material

**Table S1** The comparisons among the included study population and those adults without missing data on T2D risk markers and all adults in NHANES

| Characteristics | All adults (N=30,724) | Adults without missing values of glycemic variables (n=12878) | The present study population (n=7926) | *P^a^* | *P^b^* |
| --- | --- | --- | --- | --- | --- |
|  |  |  |  |  |  |
| Age, y, mean (SD) | 48.1 (18.6) | 48.3 (18.5) | 46.8 (18.2) | <0.001 | <0.001 |
| BMI, kg/m^2^, mean (SD) | 28.9 (7) | 28.8 (6.8) | 28.6 (6.7) | 0.005 | <0.001 |
| PIR, mean (SD) | 2.4 (1.6) | 2.4 (1.6) | 2.5 (1.6) | <0.001 | <0.001 |
| Serum cotinine, ng/mL, median (25th-75th) | 0 (0-8.7) | 0 (0-7.5) | 0 (0-4.4) | 0.02 | <0.001 |
| Sex, n(%) |  |  |  | 0.16 | 0.099 |
| Male | 14943 (48.6) | 6258 (48.6) | 3772 (47.6) |  |  |
| Female | 15781 (51.4) | 6620 (51.4) | 4154 (52.4) |  |  |
| Education levels, n(%) |  |  |  | <0.001 | <0.001 |
| <high school | 7579 (24.7) | 3160 (24.6) | 1582 (20) |  |  |
| high school | 7710 (25.1) | 3194 (24.8) | 1969 (24.8) |  |  |
| >high school | 15395 (50.2) | 6509 (50.6) | 4375 (55.2) |  |  |
| Ethnicity/Race, n(%) |  |  |  | <0.001 | <0.001 |
| Non-Hispanic White | 12529 (40.8) | 5466 (42.4) | 3733 (47.1) |  |  |
| Non-Hispanic Black | 6569 (21.4) | 2499 (19.4) | 1417 (17.9) |  |  |
| Hispanic | 3327 (10.8) | 1437 (11.2) | 782 (9.9) |  |  |
| Mexican American | 4825 (15.7) | 2056 (16) | 1181 (14.9) |  |  |
| Other Race | 3474 (11.3) | 1420 (11) | 813 (10.3) |  |  |
| Total MET score, median (25th-75th) | 1020 (0-3840) | 1080 (0-3840) | 1280 (160-4320) | <0.001 | <0.001 |
| Hypertension, n(%) |  |  |  | <0.001 | <0.001 |
| No | 18352 (59.7) | 7688 (59.7) | 5058 (63.8) |  |  |
| Yes | 12372 (40.3) | 5190 (40.3) | 2868 (36.2) |  |  |

^a^, comparison between the present study population and adults without missing data on T2D risk markers;

^b^, comparison between the present study population and all adults;

**Table S2** Multivariable-adjusted odds ratios (95% CIs)^a^ of prediabetes according to the tertiles of dietary inflammatory index. (Prediabetes was defined by other cut point (FPG 6.1-6.9 mmol/L and/or HbA1c 6.0-6.4%))

| Models | OR^b^ (95%CI) | | |  | *P* for trend | *P* for interaction ^h^ |
| --- | --- | --- | --- | --- | --- | --- |
|  | Per SD^c^ increase | T1 (-5.83, -1.04) | T2 (-1.05, 0.76) | T3 (0.77, 5.32) |  |  |
| Overall |  |  |  |  |  |  |
| Model 1 ^e^ | 1.22 (1.12, 1.32) | ref | 1.38 (1.13, 1.69) | 1.66 (1.33, 2.07) | <0.001 |  |
| Model 2 ^f^ | 1.16 (1.05, 1.28) | ref | 1.29 (1.05, 1.58) | 1.47 (1.14, 1.90) | <0.001 | **-** |
| Model 3 ^g^ | 1.12 (1.02, 1.24) | ref | 1.23 (0.99, 1.52) | 1.35 (1.03, 1.76) | <0.001 |  |
| BMI^d^ ≥30 kg/m^2^ | |  |  |  |  |  |
| Model 1 ^e^ | 1.11 (0.97, 1.27) | ref | 1.32 (0.93, 1.87) | 1.27 (0.88, 1.83) | 0.036 |  |
| Model 2 ^f^ | 1.12 (0.96, 1.31) | ref | 1.30 (0.90, 1.89) | 1.30 (0.85, 1.98) | 0.053 |  |
| Model 3 ^g^ | 1.14 (0.96, 1.35) | ref | 1.35 (0.90, 2.03) | 1.34 (0.86, 2.10) | 0.044 |  |
| BMI^d^ <30 kg/m^2^ | |  |  |  |  | 0.904 |
| Model 1 ^e^ | 1.23 (1.10, 1.37) | ref | 1.32 (1.02, 1.72) | 1.76 (1.35, 2.29) | <0.001 |  |
| Model 2 ^f^ | 1.14 (1.01, 1.29) | ref | 1.21 (0.92, 1.58) | 1.45 (1.08, 1.94) | <0.001 |  |
| Model 3 ^g^ | 1.11 (0.98, 1.26) | ref | 1.15 (0.88, 1.51) | 1.35 (1.01, 1.81) | 0.001 |  |

**Note**:

^a^ CI=confidence interval.

^b^OR=odds ratio.

^c^SD= standard deviation.

^d^BMI=body mass index.

^e^ Model 1 adjusted for energy intake (continuous) and age (continuous).

^f^ Model 2 adjusted for energy intake (continuous), age (continuous), sex (categorical), education levels (categorical), ethnicity/race (categorical), PIR (continuous).

^g^ Model 3 adjusted for energy intake (continuous), age (continuous), sex (categorical), education levels (categorical), ethnicity/race (categorical), PIR (continuous), BMI (categorical, only for overall population), hypertension (categorical), CVD (categorical), cancer (categorical), serum cotinine (continuous), and physical activity (continuous).

^h^ *P* value for interaction of the Model 3.

**Table S3** Multivariable-adjusted odds ratios (95% CIs)^a^ of diabetes according to the tertiles of dietary inflammatory index. (diabetes was defined by the cut point (FPG 126 mg/dl and/or HbA1c 6.5% and/or 2h-PG 200mg/dl))

| Models | OR^b^ (95%CI) | | |  | *P* for trend | *P* for interaction ^h^ |
| --- | --- | --- | --- | --- | --- | --- |
|  | Per SD^c^ increase | T1 (-5.83, -1.04) | T2 (-1.05, 0.76) | T3 (0.77, 5.32) |  |  |
| Overall |  |  |  |  |  |  |
| Model 1 ^e^ | 1.45 (1.28, 1.64) | ref | 2.01 (1.52, 2.65) | 2.44 (1.79, 3.33) | <0.001 |  |
| Model 2 ^f^ | 1.32 (1.17, 1.49) | ref | 1.81 (1.38, 2.37) | 1.91 (1.42, 2.58) | <0.001 | **-** |
| Model 3 ^g^ | 1.31 (1.16, 1.50) | ref | 1.68 (1.22, 2.30) | 1.78 (1.31, 2.40) | <0.001 |  |
| BMI^d^ ≥30 kg/m^2^ |  |  |  |  |  |  |
| Model 1 ^e^ | 1.29 (1.08, 1.54) | ref | 2.35 (1.39, 3.97) | 2.02 (1.22, 3.34) | 0.007 |  |
| Model 2 ^f^ | 1.27 (1.05, 1.53) | ref | 2.40 (1.44, 4.02) | 2.02 (1.20, 3.39) | 0.009 |  |
| Model 3 ^g^ | 1.28 (1.06, 1.54) | ref | 2.41 (1.43, 4.09) | 1.95 (1.17, 3.25) | 0.009 |  |
| BMI^d^ <30 kg/m^2^ |  |  |  |  |  | 0.89 |
| Model 1 ^e^ | 1.53 (1.30, 1.81) | ref | 1.56 (1.09, 2.25) | 2.55 (1.73, 3.75) | <0.001 |  |
| Model 2 ^f^ | 1.36 (1.14, 1.63) | ref | 1.31 (0.87, 1.96) | 1.86 (1.25, 2.78) | 0.003 |  |
| Model 3 ^g^ | 1.34 (1.10, 1.62) | ref | 1.22 (0.80, 1.85) | 1.72 (1.14, 2.59) | 0.013 |  |

**Note**:

^a^ CI=confidence interval.

^b^OR=odds ratio.

^c^SD= standard deviation.

^d^BMI=body mass index.

^e^ Model 1 adjusted for energy intake (continuous) and age (continuous).

^f^ Model 2 adjusted for energy intake (continuous), age (continuous), sex (categorical), education levels (categorical), ethnicity/race (categorical), PIR (continuous).

^g^ Model 3 adjusted for energy intake (continuous), age (continuous), sex (categorical), education levels (categorical), ethnicity/race (categorical), PIR (continuous), BMI (categorical, only for overall population), hypertension (categorical), CVD (categorical), cancer (categorical), serum cotinine (continuous), and physical activity (continuous).

^h^ *P* value for interaction of the Model 3.

**Table S4** The associations of dietary inflammatory index with IR and prediabetes after further adjustment for white blood cell count.

| Outcomes | OR (95%CI) | | |  |
| --- | --- | --- | --- | --- |
|  | Per SD increase | T1 (-5.83, -1.04) | T2 (-1.05, 0.76) | T3 (0.77, 5.32) |
| IR |  |  |  |  |
| All participants | 1.23 (1.14, 1.32) | ref | 1.41 (1.19, 1.68) | 1.66 (1.37, 2.01) |
| Participants with obesity | 1.18 (1.06, 1.31) | ref | 1.30 (1.03, 1.65) | 1.64 (1.26, 2.14) |
| Participants without obesity | 1.29 (1.17, 1.43) | ref | 1.58 (1.16, 2.13) | 1.71 (1.27, 2.29) |
| Prediabetes |  |  |  |  |
| All participants | 1.14 (1.06, 1.23) | ref | 1.23 (1.06, 1.43) | 1.33 (1.11, 1.61) |
| Participants with obesity | 1.19 (1.03, 1.39) | ref | 1.50 (1.12, 2.01) | 1.44 (1.00, 2.07) |
| Participants without obesity | 1.13 (1.04, 1.23) | ref | 1.15 (0.95, 1.40) | 1.32 (1.09, 1.59) |

**Note**: models were performed after adjustment for energy intake (continuous), age (continuous), sex (categorical), education levels (categorical), ethnicity/race (categorical), PIR (continuous), BMI (categorical, only for overall population), hypertension (categorical), CVD (categorical), cancer (categorical), serum cotinine (continuous), physical activity (continuous), and white blood cell count (continuous).


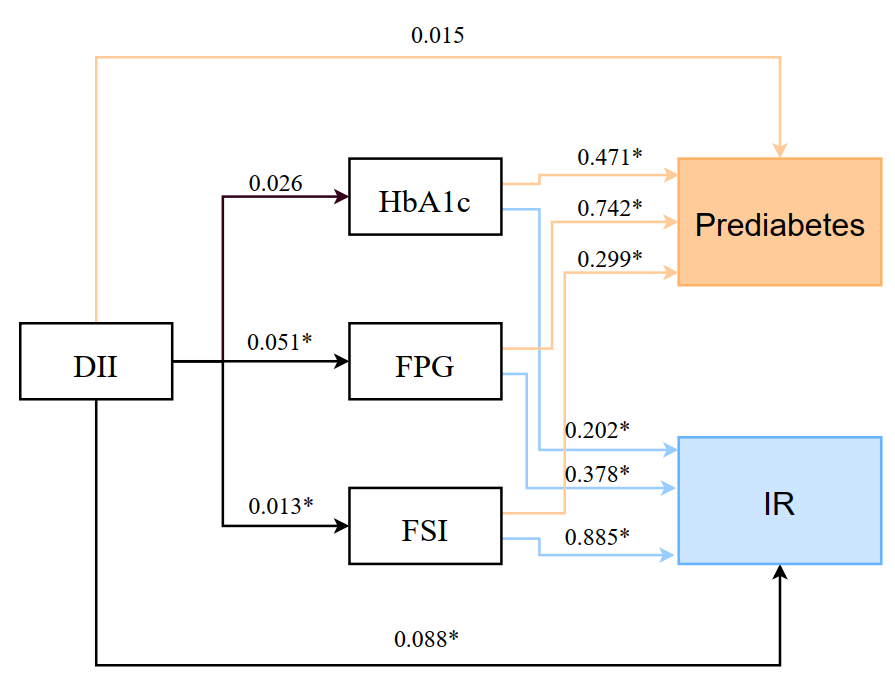


**Figure S1.** Path model of relationships of DII with IR and prediabetes.

**Note**: In this model, all variables were considered continuous, except IR and prediabetes; all relationships were adjusted for energy intake, age, sex, education levels, ethnicity/race, PIR, BMI, hypertension, serum cotinine, and physical activity. Comparative fit index (CFI) and Tucker–Lewis index (TLI) >0.9, indicating a good fit of the model.

All values in the picture were standardized coefficient.

*, *P*<0.05
